# Home-prepared food, dietary quality and socio-demographic factors: a cross-sectional analysis of the UK National Diet and nutrition survey 2008–16

**DOI:** 10.1186/s12966-019-0846-x

**Published:** 2019-09-06

**Authors:** Chloe Clifford Astbury, Tarra L. Penney, Jean Adams

**Affiliations:** MRC Epidemiology Unit & Centre for Diet and Activity Research (CEDAR), University of Cambridge, Box 285, Institute of Metabolic Science, Cambridge Biomedical Campus, Cambridge, CB2 0QQ UK

**Keywords:** Cooking, Home food preparation, Dietary quality, DASH, Socio-demographic variation, Health inequalities

## Abstract

**Background:**

Evidence suggests eating home-prepared food (HPF) is associated with increased dietary quality, while dietary quality varies across socio-demographic factors. Although it has been hypothesised that variation in HPF consumption between population sub-groups may contribute to variation in dietary quality, evidence is inconclusive. This study takes a novel approach to quantifying home-prepared food (HPF) consumption, and describes HPF consumption in a population-representative sample, determining variation between socio-demographic groups. It tests the association between HPF consumption and dietary quality, determining whether socio-demographic characteristics moderate this association.

**Methods:**

Cross-sectional analysis of UK survey data (*N* = 6364, aged≥19; collected 2008–16, analysed 2018). High dietary quality was defined as ‘DASH accordance’: the quintile most accordant with the Dietary Approaches to Stopping Hypertension (DASH) diet. HPF consumption was estimated from 4-day food diaries. Linear regressions were used to determine the association between HPF consumption and socio-demographic variables (household income, education, occupation, age, gender, ethnicity and children in the household). Logistic regression was used to determine the association between HPF consumption and DASH accordance. Interaction terms were introduced, testing for moderation of the association between HPF consumption and DASH accordance by socio-demographic variables.

**Results:**

HPF consumption was relatively low across the sample (Mean (SD) % of energy consumption = 26.5%(12.1%)), and lower among white participants (25.9% v 37.8 and 34.4% for black and Asian participants respectively, *p* < 0.01). It did not vary substantially by age, gender, education, income or occupation. Higher consumption of HPF was associated with greater odds of being in the most DASH accordant quintile (OR = 1.2 per 10% increase in % energy from HPF, 95% CI 1.1–1.3). Ethnicity was the only significant moderator of the association between HPF consumption and DASH accordance, but this should be interpreted with caution due to high proportion of white participants.

**Conclusions:**

While an association exists between HPF consumption and higher dietary quality, consumption of HPF or HPF’s association with dietary quality does not vary substantially between socio-demographic groups. While HPF may be a part of the puzzle, it appears other factors drive socio-demographic variation in dietary quality.

## Introduction

Given its substantial contribution to the ever-growing burden of chronic disease, diet has become a public health priority. Evidence suggests that higher frequency of both cooking [1–5] and eating home-prepared meals [6] is associated with an improved dietary intake.

Policymakers and advocates have stressed the importance of home food preparation, and countries such as Brazil [7], Japan [8] and Canada [9] have included cooking and food and cooking skills in their dietary guidelines. Further downstream, cooking and food classes and workshops constitute popular public health interventions [10–12]. However, systematic reviews conclude that evidence of significant and lasting change in either dietary behaviours or related health outcomes as a result of these interventions is limited [10–12].

Cooking skills interventions often target groups known to have, in general, a lower dietary quality, such as men [13] and less affluent individuals [14], suggesting that worse dietary quality in these groups is suspected to be driven by different home food preparation behaviours. An implicit assumption that some groups either cook less, or that the meals they cook are somehow less healthy, seems to underpin this sort of intervention. Cultural and behavioural differences pertaining to class, ethnicity, gender and generation could mean that the meals prepared by some groups are less healthy than others. Alternatively, home food preparation may be less important to the dietary quality of more affluent groups, as the higher purchasing power wielded by these individuals may allow them broader choice in prepared and out of home food options, including some which may be healthier. However, this remains something of an open question: while research suggests healthier diets are more expensive, studies have generally focused on the relative cost of ingredients as opposed to prepared foods [15–17].

Definition and measurement issues surround home food preparation [18, 19]. Most studies approach the issue by asking how frequently participants either make or eat a home-prepared meal [20]. Questions about how often participants prepare a meal at home target an individual behaviour, and, given the frequency of task-sharing in many households [21, 22], this question does not represent a good proxy for intake. If intake is the exposure of interest, then questions about what participants eat seem more relevant. Still, the social desirability of home-prepared food (HPF) [23, 24] may make individuals overestimate the number of home-prepared meals they consume. In addition, qualitative studies suggest that not everyone interprets terms like ‘home-prepared’ in the same way [25]. Food diaries with sufficiently detailed information might present an opportunity to derive a more ‘objective’, or, at least, internally consistent, measure of HPF consumption.

This study will answer the questions:

1. What is the proportion of total energy derived from HPF in the UK population, and does this vary by socio-demographic characteristics?
2. Is proportion of total energy derived from HPF associated with diet quality?
3. Do socioeconomic position and demographic variables moderate the relationship between the energy derived from HPF and dietary quality?

## Methods

This study represents a cross-sectional analysis of dietary surveillance data from the UK National Diet and Nutrition Survey (NDNS) 2008–16 (May 2018 release) [26]. It is reported according to the STROBE-nut recommendations [27].

NDNS is an annual cross-sectional survey which collects information on food consumption and nutritional and health status of free-living individuals in the UK. Sampling, recruitment and data collection are carried out in a consistent manner, allowing data from different survey years to be combined for cross-sectional analysis. A detailed account of the NDNS recruitment and sampling protocol has been published elsewhere [28–30]. Individuals aged ≥19 years at the time of participation who completed three or 4 days of the food diary were included in the analyses.

### Dietary assessment

Participants completed unweighed food diaries, including all food and beverages consumed both inside and outside the home. This process is described in detail elsewhere [31]. Participants also recorded where the food was eaten, for example at home, in a restaurant or café, or at work. This variable included a specific category for food eaten at work but brought from home.

### Characterisation of food-related variables

As previously, food items listed in food diaries were classified by the authors as either requiring or not requiring home preparation [32]. All foods were classified as home-prepared except those listed in Table 1. Foods which should not be classified as being home-prepared were decided by the authors a priori.

**Table 1:** Foods not classified as home-prepared

Foods prepared and eaten outside the home (e.g. food eaten in a restaurant or café) Foods prepared outside the home and eaten in the home (e.g. takeaway and delivery foods) Foods eaten as purchased (e.g. crisps, sweets, granola bars, juice and soft drinks, store-bought sandwiches, prepared and whole pieces of fruit) Foods requiring the application of heat or the addition of hot water but no other preparation (e.g. frozen and refrigerated ready meals, tinned soup, instant noodles, instant oats)	

Definitions of ‘cooking’ have been discussed extensively and remain contested [18, 33, 34], with many definitions not deeming the application of heat to be a necessary part of this process [34, 35]. As a result ‘home food preparation’ and ‘home-prepared food’ seem more accurate and are the concepts deployed here. Different, but related, conceptualisations exist, such as food ‘prepared from scratch’ [36]. or food that is not ‘from outside the home’ [37]. The conceptualisation of HPF used here reflects several conceptions of ‘cooking’, or home food preparation, drawn from qualitative studies [38, 39] as well as behaviours which are habitually enquired about in studies of ‘cooking’, such as blending, mixing, boiling, chopping, roasting and pan frying [19]. From this conceptualisation of home food preparation, a set of behaviours, we defined foods which we would deem to be home-prepared as being the products of these behaviours.

**Fig. 1:**
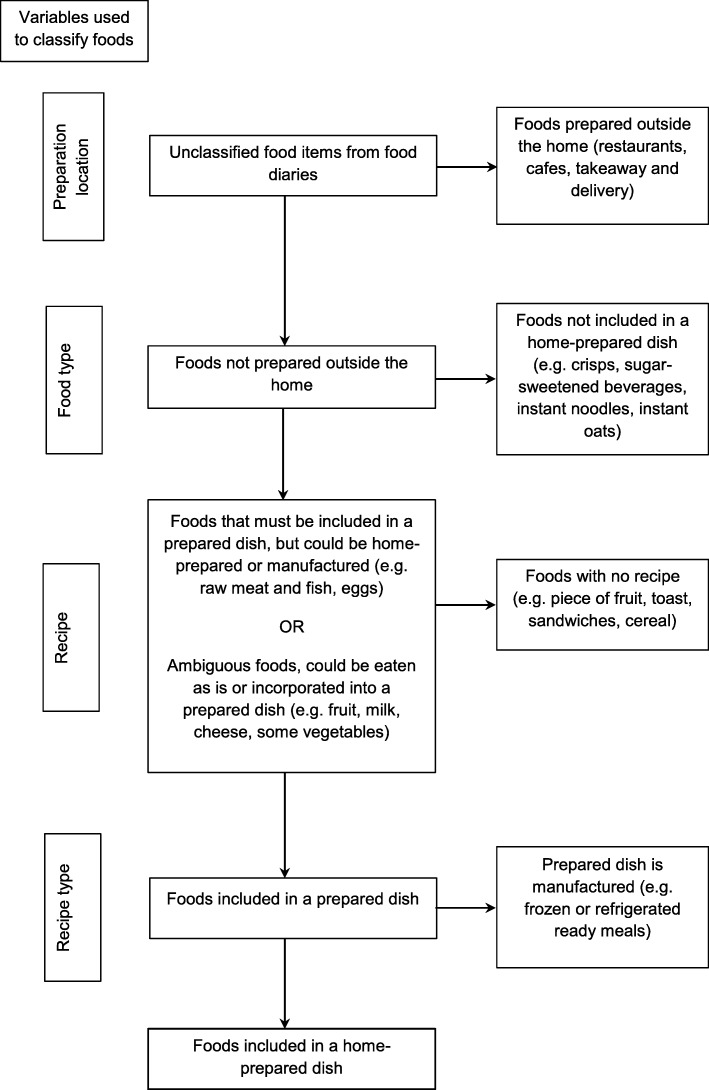
Flow diagram for classification of foods as being home-prepared

Food classification was carried out using food diary variables as illustrated in Fig. 1, with foods which were not classified as home-prepared being successively removed until only food included in home-prepared dishes remained. The proportion of energy from HPF was then calculated for each participant by summing the energetic content of foods classified as home-prepared and dividing them by the participant’s total energy intake.

Dietary quality was determined by quantifying accordance to the Dietary Approaches to Stopping Hypertension (DASH) dietary pattern using a method adapted for use with NDNS [40] from an existing index [41]. The DASH diet has been shown to lower blood pressure [42] and reduce low-density lipoprotein cholesterol levels [42], as well as being associated with a lower risk of stroke and coronary heart disease [41]. This score is based on food and nutrients emphasised or minimised in the DASH diet, and has eight components: high intake of fruits, vegetables, nuts and legumes, low-fat dairy products, and whole grains; and low intake of sodium, red and processed meats, and non-extrinsic milk sugars; all adjusted for total energy intake. The score is adjusted for overall energy intake. Components are evenly weighted, and three components (sodium, sugar, and red and processed meats) are reverse-scored, so that higher consumption would lower an individual’s DASH score. The overall score ranges between 8 and 40, with higher scores indicating a diet which has greater accordance with the DASH pattern.

This study models DASH accordance as a binary variable, with participants in the top quintile of DASH score being considered the most DASH-accordant, a method which has been previously employed by a number of studies [40, 43, 44].

### Socio-demographic variables

Age, sex, ethnicity, and the presence of children in participant households were determined using self-reported survey responses. Socioeconomic position was also assessed using self-reported survey responses, and was characterised using three markers: occupation (among employed participants; occupation was classified using the simplified three-class version of the National Statistics Socio-economic Classification described by the UK’s Office for National Statistics [45]), highest educational attainment, and quintile of annual household income equivalised for household composition. Evidence suggests these socioeconomic markers present different associations with dietary intake, and are not necessarily interchangeable [46].

### Analysis

Analysis was conducted in 2018. Variables were weighted using weights provided by the NDNS study team, which sought to mitigate bias resulting from the survey design and from differential non-response by individual participants [47].

The mean proportion of energy from HPF consumed by participants was determined. Linear regression was used to determine how this proportion varied by socio-demographic characteristics, using socio-demographic characteristics as exposure variable and proportion of energy from HPF as an outcome variable.

Logistic regression was used to determine the association between proportion of energy from HPF and DASH accordance. Interaction terms were introduced to test for effect modification by socio-demographic characteristics. If any interaction terms were significant, models stratified by the socio-demographic variable in question were run to determine association between energy from HPF and DASH adherence in each population sub-group.

All regressions were mutually adjusted for all socio-demographic variables. All analyses were conducted using Stata (version 14; Stata Corp.). Alpha-level of 0.01 was used throughout to test for statistical significance in order to compensate for multiple testing.

## Results

Overall, 54% (*N* = 12,070) of individuals selected to take part in NDNS provided useable food diaries (three or four complete days), including 6364 participants aged ≥19 years [28, 29, 48].

The mean percentage of energy derived from HPF in the sample was relatively low (Mean (SD) = 26.5%(12.1%)). Table 2 describes the proportion of energy derived from HPF by population sub-group, and presents the results of a linear regression with socio-demographic characteristics as the exposures and proportion of energy from HPF as the outcome.

**Table 2:** Description of energy from home-prepared food by population sub-group, and associations between socio-demographic characteristics and proportion of energy from home-prepared food

Characteristic	n (%)	Proportion of energy from home-prepared food (%)			
		Mean (SD) (%)	Regression^a^ coefficient	95% CI	P > |t|
*Total*	6364 (100)	26.5 (12.1)			
Age group					
*19–24 (ref.)*	645 (10.1)	26.6 (13.0)			
*25–49*	2761 (43.4)	27.0 (12.5)	−0.2	−2.2-1.8	0.84
*50–64*	1547 (24.3)	26.2 (11.8)	−0.1	−2.3-2.0	0.93
65+	1411 (22.2)	25.8 (11.1)	0.3	−1.9-2.5	0.81
Sex					
*Male (ref.)*	2640 (41.5)	25.8 (12.1)			
*Female*	3724 (58.5)	27.1 (12.1)	1.5	0.6–2.4	**< 0.01**
Ethnicity					
*White (ref.)*	5907 (92.9)	25.9 (11.6)			
*Mixed ethnicity*	58 (0.9)	28.0 (13.5)	−0.9	−5.7-3.9	0.70
*Black or Black British*	133 (2.1)	37.8 (15.8)	14.5	10.9–18.2	**< 0.01**
*Asian or Asian British*	177 (2.8)	34.4 (14.9)	7.6	4.8–10.3	**< 0.01**
*Other*	82 (1.3)	34.6 (14.4)	10.8	6.4–15.1	**< 0.01**
Children living at home					
*None (ref.)*	4392 (69.0)	26.0 (11.9)			
*Children aged < 16*	1103 (17.3)	27.5 (12.2)	0.2	−1.1-1.8	0.71
*Children aged < 5*	869 (13.7)	28.3 (12.7)	1.7	0.2–2.5	0.03
Educational attainment					
*Degree level (ref.)*	1461 (25.5)	27.8 (12.2)			
*12–13 years of education*	1505 (26.2)	26.4 (11.8)	−1.7	−2.9- -0.4	**< 0.01**
*11 years of education and/or vocational course*	1315 (22.9)	25.9 (11.9)	−1.0	−2.4-0.4	0.18
*< 11 years of education*	1457 (25.4)	25.6 (12.0)	−2.5	−4.1- -0.9	**< 0.01**
Equivalised income quintile					
*5 (Highest) (ref.)*	1061 (19.5)	26.4 (11.9)			
*4*	1093 (20.1)	26.6 (11.4)	0.8	−0.5-2.2	0.21
*3*	1099 (20.2)	26.7 (12.6)	1.8	−0.3-3.2	0.02
*2*	1067 (19.6)	26.0 (12.5)	0.9	−0.6-2.4	0.25
*1 (Lowest)*	1132 (20.8)	26.8 (12.2)	1.2	−0.4-2.8	0.15
Occupation					
*Professional and managerial (ref.)*	2468 (40.7)	26.5 (11.6)			
*Intermediate occupation*	1911 (31.5)	26.7 (11.9)	0.8	−0.2-1.9	0.13
*Routine and manual occupation*	1684 (27.8)	28.6 (12.6)	0.2	−1.1-1.4	0.79

Boldface indicates statistical significance (*p* < 0.01)

^a^Mutually adjusted for other socio-demographic variables

Proportion of energy from HPF did not vary substantially by socio-demographic variables. A small increase was associated with being female v male (27.1 v 25.8%, *p* < 0.01), and a small decrease was associated with having 12–13 years of education or < 11 years of education relative to having a university degree (26.4 p < 0.01 and 25.6 p < 0.01 v 27.8% respectively). More substantial variation was associated with ethnicity, with Black participants (37.8%), Asian participants (34.4%) and participants belonging to other ethnic groups (34.6%) consuming substantially more HPF than White participants (v 25.9%, all *p* < 0.01).

Meanwhile, the expected associations between socio-demographic characteristics and dietary quality were found (methods and results reported in Appendix 2).

Table 3 shows the results of a logistic regression with proportion of energy from HPF as the exposure and DASH adherence as the outcome before and after adjustment for age, sex, ethnicity, presence of children in the household, income, education and occupation (full reporting of adjusted model in Appendix 1). In the unadjusted model, there is a small but statistically significant association between the variables, with an increase in 10% of energy from HPF resulting in a 20% increase in the odds of being DASH-adherent. This remained unchanged after adjustment. Given the low mean value of energy from HPF, a 10% increase would represent a substantial change, slightly lower than a change of one standard deviation (12.1%).

**Table 3:** Logistic regression of DASH adherence and proportion of energy from home-prepared food (per 10%)

Model	OR	95% CI	P > |t|
Unadjusted model	1.19	1.13–1.27	**< 0.01**
Adjusted model^a^	1.20	1.11–1.31	**< 0.01**

Boldface indicates statistical significance (*p* < 0.01)

^a^Mutually adjusted for other socio-demographic variables

The interaction term for Asian participants relative to White participants was significant (*p* < 0.01), suggesting the association between proportion of energy from HPF and DASH adherence was different in this group. Although the interaction term for Asian ethnicity was statistically significant, stratified regression was not performed. Due to the small number of non-White participants in the NDNS sample (see Table 2), the interpretation of the interaction term was challenging, and running fully adjusted logistic regressions for each sub-group was impossible. While there may be a difference in the association between HPF consumption and DASH accordance in different ethnic groups, a more ethnically diverse sample would be required to properly examine it.

All other interaction terms were non-significant (*p* > 0.01); further analyses were therefore not performed.

## Discussion

This study took a novel approach to quantifying HPF consumption, deriving estimates from 4-day food diaries. The proportion of energy from HPF was relatively low across the sample (Mean (SD) = 26.5%(12.1%)). Consumption of HPF did not vary substantially by any of the socio-demographic variables considered here, with the exception of ethnicity. Meanwhile, dietary quality varied extensively across socio-demographic variables, in ways similar to what has been seen in other studies, with women, older participants, more affluent participants and non-white participants displaying higher dietary quality than their counterparts.

An association between HPF consumption and dietary quality appeared across the sample: a 10% increase in energy derived from HPF was associated with a 20% increase in the odds of falling in the most DASH-accordant quintile. However, it must be acknowledged that a 10% increase is large given the low contribution of HPF to the energetic intake of most participants (close to one standard deviation, at 12%). Socio-demographic variables did not moderate the association between consumption of HPF and dietary quality, except potentially in the case of ethnicity.

Non-White participants consumed a greater proportion of energy from HPF, and had a higher dietary quality. In addition, moderation analysis suggested that the association between consumption of HPF and dietary quality may differ across ethnicities. However, it is difficult to ascertain this: small numbers in other ethnic groups precluded stratified analysis. This could be investigated through further research.

Weighted, NDNS is UK population-representative, giving this study broader generalisability. However, a similar analysis conducted in different national contexts might yield different results, particularly in countries where ‘traditional’ food patterns remain stronger than they seem in the UK, such as in countries where a substantial proportion of the population adheres to the Mediterranean diet pattern. Comparative research of, for example, the UK and France suggests that, while there are certain convergent patterns that emerge in both countries, such as an increased use of convenience foods, and a reporting of a lack of time to cook, there are also ways in which home food practices remain distinct between countries, such as the absence of totally pre-prepared ready meals among French participants, and an increased propensity to cook ‘from scratch’ [49]. Meanwhile, a comparative analysis of trends in time spent eating at home in five different countries found that time spent decreased in all countries except France [50]. It would be interesting to see how the association found here might differ in a range of contexts where food practices might diverge.

This study uses the DASH score, a well-evidenced and relatively comprehensive measure of dietary quality. The food-related variables in this study were derived from unweighed, self-reported food diaries. While evidence suggests that food diaries are a more accurate measure of dietary intake than other common measures such as food frequency questionnaires [51], misreporting in self-measured dietary instruments is a well-documented limitation [48, 52].

In addition, there is potential for residual confounding due to characteristics that were not adjusted for in this analysis, such as food insecurity or characteristics of the food environment. Although there is evidence that both of these factors are associated with dietary quality, the evidence on how they are related to home food preparation is more limited. One study of home food preparation in low-income, food insecure women in Canada found that households that were more food insecure reported less complex home food preparation, though not less frequent preparation of meals ‘from scratch’ [53], although it is not clear whether this is suggestive of a protective effect of home food preparation against food insecurity, or a decrease in home food preparation in response to the stresses attendant on becoming food insecure, or some further factor. Regarding food environments, a study set in urban regions across five European countries (including the UK) found that greater access to restaurants was associated with reduced self-reported frequency of cooking [54]. Both these exposures are also likely to be socio-economically patterned, and may associate with some of the socio-economic indicators examined here. Further work could consider how they might affect the association between HPF consumption and dietary quality.

Finally, this analysis represents a cross-sectional analysis of the associating between HPF consumption and dietary quality. Further, longitudinal work could be done to verify how HPF consumption relates to diet-related health outcomes.

The relatively low proportion of energy from HPF is reflective of our measure: many common breakfast choices (such as toast or cereal) and lunch choices (sandwiches) are not classified as home-prepared. While our choices regarding classification could be debated, our measure has the advantage of internal consistency, with the definition of what is home-prepared being the same for all participants. In addition, our classification is informed by the literature, reflecting qualitative conceptualisations [33, 39] and behavioural measures used in quantitative studies of home food preparation [19].

Many studies of dietary quality and food preparation have focused on home food preparation frequency [1, 36, 55–59], and skills [56, 60–63] as opposed to HPF consumption. Some studies of HPF consumption and dietary quality exist, but it is difficult to compare results due to the diversity of measures of dietary quality in use. One study using a UK-based cohort examined the association between self-reported frequency of consuming home-prepared meals and several indices of dietary quality, including DASH score [6], estimating that eating a home-prepared main meal more than five times a week, as opposed to less than three times a week, was associated with an 0.61 increase in DASH score. Due to the relative nature of the DASH index used here [41], and the different approaches to modelling both DASH score and consumption of HPF, it is difficult to carry out an exact comparison, other than to say that both associations are statistically significant but moderate.

Quantitative studies of HPF consumption and socio-demographic variables are limited, although analyses of home preparation skill and frequency do exist [64–66]. Studies generally find that women cook more frequently than men [64, 65], which may also be the case in this dataset. Two studies from the United States found households with lower household income and educational attainment were more likely to cook always or never, compared to more affluent households who were more likely to sometimes cook at home. [1, 67] These analyses also found that Black households reported cooking less frequently, whereas the reverse is suggested by our data. However, the different historical, cultural and national origins of Black populations in the US and the UK make distinct dietary patterns unsurprising. Black British populations are dominated by individuals of Caribbean and West African ancestry, communities themselves have distinct dietary patterns [68], despite being grouped together within this study due to limited ethnic diversity in our study sample.

These results confirm an association between HPF consumption and dietary quality, although the association is relatively small. As interventions to increase home food preparation encounter issues of cost and scalability, as well as showing equivocal evidence of long-term impact in participants [10–12], it is unclear that this justifies further policy action in terms of improving dietary quality. Our previous work suggests that it is possible to eat healthily while consuming very little HPF [32]; while an association with home food preparation exists, so may other behavioural routes to high dietary quality. In addition, the small contribution of HPF to the energetic intake of most participants suggests that changing home food preparation practices might have more limited potential to impact overall dietary quality than might be assumed.

These results further suggest that differences in levels of consumption of HPF may not be key drivers of dietary inequalities along the socio-demographic axes examined here, and although this could be further explored, it does not appear that HPF consumption mediates the association between socio-demographic factors and dietary quality.

In addition, most socio-demographic variables do not appear to moderate the association between consumption of HPF and dietary quality, suggesting that different groups are eating HPF with similar nutritional properties, although other dietary components may be compensating in some systematic way.

Overall, it appears that neither the amount nor the nature of HPF consumed by different population sub-groups is contributing substantially to the inequalities in dietary quality known to exist across these groups (and demonstrated again in this data). One exception to this may be in the case of variation across ethnicities, although the nature of this sample makes this difficult to comment upon.

This study presents a comparison between a nutrition-based characterisation of diet, DASH accordance, and a behaviour-based one, consumption of HPF. Other behaviour-based characterisations of diet exist, such as food ‘cooked from scratch’ or ‘traditional recipes’. More might be developed through qualitative work delving into how individuals conceptualise the food they prepare and eat. In order to understand which behaviours are most important for dietary quality, it is worth continuing to think about diet not only in nutritional terms but in behavioural ones reflecting people’s daily practices, and understanding how these drive dietary intake.

Although consumption of HPF shows a small association with dietary quality, it does not appear to drive dietary inequalities between population sub-groups. This suggests that the remaining components of the diet, food consumed outside the home, and food consumed at home that is not home-prepared, may be driving dietary inequalities, which could be examined through further research. Some interventions have already sought to target these food sources, including supermarket interventions aiming to promote purchases of healthier snacks [69], and restaurant menu labelling providing information on the nutrition and energetic content of various dishes [70].

## Conclusion

This study suggests relatively low levels of consumption of HPF across the population-representative sample, and confirms a statistically significant but moderate association between consuming HPF and dietary quality. In addition, neither the amount nor the type of HPF consumed appeared to contribute substantially to inequalities in dietary quality across population sub-groups. These results suggest that the potential of changing HPF consumption as a means of improving dietary quality overall, and particularly for addressing diet-driven health inequalities, may be relatively limited. Further research may help to determine which other dimensions of food practices make a more substantial contribution to dietary quality and dietary inequalities.

## Data Availability

The datasets supporting the conclusions of this article are available in the UK Data Service repository, 10.5255/UKDA-SN-6533-8 https://discover.ukdataservice.ac.uk/catalogue/?sn=6533.

## Appendix 1

### Association between home-prepared food consumption and DASH accordance

**Table 4:** Association between home-prepared food consumption and DASH accordance. Full adjusted model of the association between home-prepared food consumption and DASH accordance

Explanatory variables	OR^a^	95% CI	P > |t|
Age group			
*19–24 (ref.)*			
*25–49*	3.2	1.5–6.9	**< 0.01**
*50–64*	8.2	3.8–17.6	**< 0.01**
65+	9.9	4.6–21.3	**< 0.01**
Sex			
*Male (ref.)*			
*Female*	1.6	1.4–2.0	**< 0.01**
Ethnicity			
*White (ref.)*			
*Mixed ethnicity*	1.8	0.6–4.7	0.27
*Black or Black British*	1.3	0.7–2.5	0.38
*Asian or Asian British*	4.6	2.8–7.4	**< 0.01**
*Other*	1.0	0.5–2.0	0.94
Children living at home			
*None (ref.)*			
*Children aged < 16*	1.0	0.7–1.3	0.97
*Children aged < 5*	0.7	0.5–1.0	0.04
Educational attainment			
*Degree level (ref.)*			
*12–13 years of education*	0.6	0.5–0.8	**< 0.01**
*11 years of education and/or vocational course*	0.5	0.4–0.7	**< 0.01**
*< 11 years of education*	0.3	0.2–0.4	**< 0.01**
Equivalised income quintile			
*5 (Highest) (ref.)*			
*4*	0.9	0.7–1.1	0.24
*3*	0.7	0.5–0.9	0.02
*2*	0.7	0.5–1.0	0.04
*1 (Lowest)*	0.6	0.4–0.9	**< 0.01**
Occupation			
*Professional and managerial (ref.)*			
*Intermediate occupation*	0.7	0.5–0.9	**< 0.01**
*Routine and manual occupation*	0.7	0.6–1.0	0.05
Energy from home-prepared food	1.2	1.1–1.3	**< 0.01**

Boldface indicates statistical significance (*p* < 0.01)

^a^Mutually adjusted for other socio-demographic variables

## Appendix 2

### Variation in dietary quality by socio-demographic characteristics

Table 5 shows the results of a logistic regression with socio-demographic characteristics as the exposure and classification in the top quintile for DASH accordance as the outcome.

**Table 5:** Associations between DASH accordance and socio-demographic characteristics

Characteristic	n (%)	Relative DASH accordance			
		Proportion DASH-accordant (%)	OR^a^	95% CI	P > |t|
*Total*	6364 (100)	19.8			
Age group					
*19–24 (ref.)*	645 (10.1)	5.0			
*25–49*	2761 (43.4)	16.7	3.2	1.5–6.8	**< 0.01**
*50–64*	1547 (24.3)	28.1	8.1	3.9–17.2	**< 0.01**
65+	1411 (22.2)	23.3	9.9	4.7–21.0	**< 0.01**
Sex					
*Male (ref.)*	2640 (41.5)	16.0			
*Female*	3724 (58.5)	22.5	1.7	1.4–2.1	**< 0.01**
Ethnicity					
*White (ref.)*	5907 (92.9)	18.9			
*Mixed ethnicity*	58 (0.9)	31.0	1.7	0.6–4.7	0.32
*Black or Black British*	133 (2.1)	27.1	1.7	0.9–3.2	0.09
*Asian or Asian British*	177 (2.8)	39.6	5.1	3.2–8.2	**< 0.01**
*Other*	82 (1.3)	24.4	1.3	0.6–2.5	0.51
Children living at home					
*None (ref.)*	4392 (69.0)	21.5			
*Children aged < 16*	1103 (17.3)	18.4	1.0	0.7–1.3	0.99
*Children aged < 5*	869 (13.7)	13.2	0.7	0.5–1.0	0.07
Educational attainment					
*Degree level (ref.)*	1461 (25.5)	33.6			
*12–13 years of education*	1505 (26.2)	19.3	0.6	0.5–0.8	**< 0.01**
*11 years of education and/or vocational course*	1315 (22.9)	15.1	0.5	0.4–0.7	**< 0.01**
*< 11 years of education*	1457 (25.4)	13.0	0.3	0.2–0.4	**< 0.01**
Equivalised income quintile					
*5 (Highest) (ref.)*	1061 (19.5)	30.0			
*4*	1093 (20.1)	24.3	0.9	0.7–1.1	0.32
*3*	1099 (20.2)	17.8	0.7	0.5–1.0	0.03
*2*	1067 (19.6)	16.2	0.7	0.5–1.0	0.05
*1 (Lowest)*	1132 (20.8)	12.8	0.6	0.5–0.9	**< 0.01**
Occupation					
*Professional and managerial (ref.)*	2468 (40.7)	27.8			
*Intermediate occupation*	1911 (31.5)	17.1	0.7	0.6–0.9	**< 0.01**
*Routine and manual occupation*	1684 (27.8)	12.0	0.8	0.6–1.0	0.06

Boldface indicates statistical significance (*p* < 0.01)

^a^Mutually adjusted for other socio-demographic variables

As in previous studies, DASH accordance varied extensively by demographic variables, with older people (OR 9.9(95% CI 4.7-21.0) for participants aged 65 and over relative to participants aged 19-24), women (OR 1.7(95% CI 1.4-2.1) relative to men) and Asian participants (OR 5.1 (95% CI 3.2-8.3) relative to white participants) being significantly more likely to be in the most DASH-accordant quintile. Participants with a lower educational attainment were less likely to be in the top quintile (OR 0.3 (95% CI 0.2-0.4) for participants with less than 11 years of education relative to participants with a degree-level education), as were participants in the lowest quintile of household income (OR 0.6 (95% CI 0.5-0.9) relative to top income quintile). Participants in intermediate roles were less likely to be DASH-accordant than their counterparts in professional or managerial roles (OR 0.7(95% CI 0.6-0.9)).
